# Graphene–Selenium Hybrid Microballs as Cathode Materials for High-performance Lithium–Selenium Secondary Battery Applications

**DOI:** 10.1038/srep30865

**Published:** 2016-08-02

**Authors:** Hee-Chang Youn, Jun Hui Jeong, Kwang Chul Roh, Kwang-Bum Kim

**Affiliations:** 1 Department of Materials Science and Engineering, Yonsei University, Seoul 120-749, Republic of Korea; 2 Energy and Environmental Division, Korea Institute of Ceramic Engineering and Technology, Jinju 660-031, Republic of Korea

## Abstract

In this study, graphene–selenium hybrid microballs (G–SeHMs) are prepared in one step by aerosol microdroplet drying using a commercial spray dryer, which represents a simple, scalable continuous process, and the potential of the G–SeHMs thus prepared is investigated for use as cathode material in applications of lithium–selenium secondary batteries. These morphologically unique graphene microballs filled with Se particles exhibited good electrochemical properties, such as high initial specific capacity (642 mA h g^−1^ at 0.1 C, corresponding to Se electrochemical utilisation as high as 95.1%), good cycling stability (544 mA h g^−1^ after 100 cycles at 0.1 C; 84.5% retention) and high rate capability (specific capacity of 301 mA h g^−1^ at 5 C). These electrochemical properties are attributed to the fact that the G–SeHM structure acts as a confinement matrix for suppressing the dissolution of polyselenides in the organic electrolyte, as well as an electron conduction path for increasing the transport rate of electrons for electrochemical reactions. Notably, based on the weight of hybrid materials, electrochemical performance is considerably better than that of previously reported Se-based cathode materials, attributed to the high Se loading content (80 wt%) in hybrid materials.

Rapid development in electric vehicles as well as large-scale renewable energy storage devices has resulted in an urgent need for lithium secondary batteries with high energy densities, power densities, long cycling lives and low cost. Currently available lithium-ion batteries (LIBs) have been considered for use in electric automobiles. However, despite extensive efforts focused on the development of LIBs, the highest energy storage capacity exhibited by LIBs is not sufficient for meeting the demands of electric automobiles^1^,^2^. In this regard, recently, lithium secondary batteries fabricated using group 6A elements, such as sulfur and selenium, as cathode materials and metallic lithium as anode material have attracted considerable attention, attributed to their ultrahigh energy storage capacities^3^,^4^,^5^. Elemental Se is considered to be a potential candidate as cathode material for high-energy rechargeable lithium batteries, even though research on Li–Se batteries is still in the nascent stage. Although Se exhibits a theoretical gravimetric capacity (675 mA h g^−1^) less than that of sulfur (1675 mA h g^−1^), its higher density (4.82 g cm^−3^; ca. 2.5 times greater than that of sulfur) compensates for its low gravimetric capacity and results in a volumetric capacity as high as 3253 mA h cm^−3^, which is comparable to that of sulfur (3467 mAh cm^−3^)^6^. Moreover, its electronic conductivity (σ_Se_ = 1 × 10^−3 ^S m^−1^) is considerably greater than that of sulfur (σ_S_ = 5 × 10^−28 ^S m^−1^), suggesting that the use of Se results in higher utilisation of electrochemically active materials and a more rapid reaction with lithium ions^6^,^7^. Nevertheless, the use of Se as a cathode material involves significant challenges, namely 1) intermediate selenium compounds (i.e. polyselenides) generated during charging/discharging readily dissolve in organic electrolytes, which shuttle to the anode side, resulting in poor cycling stability^8^,^9^. For overcoming this issue, several approaches have been reported, such as impregnating selenium into porous carbon, ensuring the adsorption of polyselenides on porous metal oxides and inserting carbon layers between the separator and cathode^6^,^10^,^11^,^12^,^13^. Another effective approach is to use graphene as a polyselenide confinement matrix as well as an electrically conductive material. Graphene as an excellent template material can be combined with particles of group 6A elements, such as S, for preventing the dissolution of intermediate species (such as polysulfides), thereby resulting in the formation of an electrical path^14^,^15^,^16^. However, few studies have used graphene as polyselenide confinement matrices, as well as an electrically conductive agent, in Li–Se rechargeable batteries.

In this study, morphologically unique graphene–selenium hybrid microballs (G–SeHMs), with the highest loading of Se (80 wt%) reported thus far, to the best of our knowledge, were fabricated for use as cathode material in Li–Se rechargeable batteries. Graphene sheets are used for encapsulating micro-sized Se particles in a form of microballs by aerosol microdroplet drying method, which is a simple, scalable continuous process for manufacturing hybrid materials^17^. Well-encapsulated Se-based hybrid microballs by graphene sheets serve as confinement matrices for suppressing the dissolution of polyselenide into the organic electrolyte during charging/discharging, as well as provide an electrically conducting path for increasing the electron transport rate. Thus, these hybrid materials as cathode in Li–Se rechargeable batteries exhibit a high specific capacity, good rate capability and stable cycling performance. Notably, with high loading of Se in this hybrid cathode material, its electrochemical performance based on the weight of hybrid materials is remarkably better than that reported previously for Se-based cathode materials.

## Results

**Figure 1:**
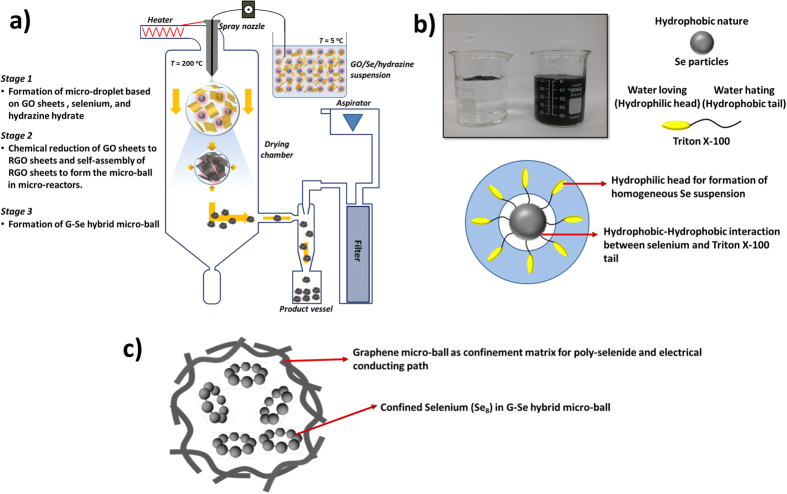
Schematic of (**a**) process employed for synthesizing the graphene–selenium hybrid microballs (denoted as G–SeHMs), involving the use of a commercial spray dryer for drying aerosol microdroplets of the GO/surfactant-decorated Se/hydrazine hydrate suspension, (**b**) formation of a stable Se suspension by the addition of Triton X-100 as a non-ionic surfactant and photographs of the colloidal Se suspension without/with Triton X-100 and (**c**) the proposed morphology of G–SeHMs.

Figure 1(a) shows the synthesis of G–SeHMs as a cathode material for applications to Li–Se secondary batteries; synthetic details have been provided in the experimental section. For synthesizing G–SeHMs by aerosol microdroplet drying, a stable aqueous colloidal suspension of graphene oxide (GO) and Se particles is typically prepared. However, as Se particles are hydrophobic, the particles are not readily dispersed in an aqueous system. Thus, Triton X-100, a nonionic surfactant, is added for altering the surface chemistry of the particles so as to prepare a stable Se colloidal suspension in water. As shown in Fig. 1(b), the Triton-X-100-decorated Se particles were readily dispersed in the aqueous system. Furthermore, hydrazine hydrate was added as the chemical reducing agent in the as-prepared aqueous suspension. Nonconductive GO sheets are well known to be easily converted into sheets of electrically conductive reduced GO (RGO) by chemical reduction using hydrazine hydrate at high temperature^18^. Hence, hydrazine hydrate is added in the as-prepared aqueous suspension as a precursor solution, namely, so as to chemically reduce the nonconductive GO sheets during spray-drying. The aqueous suspension should be maintained at low temperature (below 5 °C) for inhibiting the reduction of the nonconductive GO sheets before the start of spray-drying. Otherwise, (i.e. maintained at room temperature), the stability of the aqueous suspension is literally destroyed as the hydrophilic GO sheets easily convert into hydrophobic RGO sheets. The stable colloidal suspension containing the GO sheet, surfactant-decorated Se particles and hydrazine hydrate in an aqueous system was atomised using a spray nozzle, resulting in aerosol microdroplets; these microdroplets were sprayed downwards towards a heated zone at 200 °C, which is considerably greater than the boiling point of water. As the microdroplets passed through the heated zone, the water in the microdroplets evaporated with the simultaneous chemical reduction of the nonconductive GO sheets, resulting in conductive RGO sheets. When water completely evaporated, the Se particles were encapsulated in the RGO microballs. Subsequently, the final product was washed with ethanol and acetone for removing the residual Triton X-100. Tdy in shown in Fig. 1(c); the graphene microballs are expected to serve as a confinement matrix for dissolving polyselenide in the organic electrolyte, as well as an electron conduction path.

**Figure 2:**
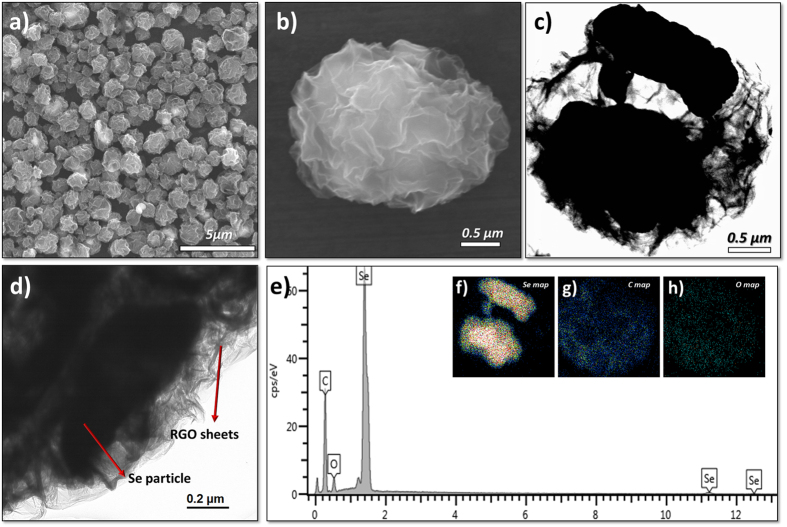
Morphological characteristics of G–SeHMs. (**a**) Low- and high-magnification SEM images. (**c**) Low- and (**d**) high-magnification HRTEM images. (**e**) Elemental spectrum, and elemental maps of (**f**) selenium, (**g**) carbon and (**h**) oxygen shown in the insets.

Figure 2 shows the morphological characteristics of the G–SeHMs thus obtained, as recorded by scanning electron microscopy (SEM), high-resolution transmission electron microscopy (HR-TEM) and energy-dispersive X-ray spectroscopy (EDX). As shown in Fig. 2(a), 2–3 μm uniform-sized G–SeHMs were successfully prepared by aerosol microdroplet drying from a stable colloidal suspension containing GO/surfactant-decorated Se/hydrazine hydrate. Furthermore, wrinkled two-dimensional RGO sheets with a lateral size of a few micrometres (see Fig. S1 in Supporting Information) formed complete microballs (Fig. 2(b)), suggesting that all Se particles are encapsulated within these microballs. For observing the interior of G–SeHMs in detail (Fig. 2(c–h)), HR-TEM imaging and elemental EDX mapping were conducted. The TEM images in Fig. 2(c,d) show the local structure of the G–SeHMs, where crumpled RGO sheets (light contrast) were found to be evenly dispersed outside the microball and Se particles (dark contrast) were physically confined well in the RGO microball, respectively. Se particles with an average size of 1 μm, same as that of the commercial Se particles used in this study, were observed within the G–SeHMs; this observation was also confirmed by point- and line-scanning spectroscopy and elemental SEM mapping (Fig. S2 in Supporting Information). As shown in the high-magnification TEM image in Fig. S3 (a) (Supporting Information), ultra-thin layers were observed at the edges, suggesting a single to few layers of graphene in the RGO microball. Furthermore, as can be observed in the SAED pattern shown in the inset of Fig. S3 (b), well-defined diffraction spots were observed in the hexagonal pattern, indicating that the basal plane in the RGO microball mostly consists of sheets with a single layer or a few layers comprising honeycomb carbon networks. For investigating the structural features of the interior of the RGO microball, we used a synthetic process identical to that for G–SeHMs for preparing an RGO microball without Se particles and characterised the microball by cross-sectional SEM and TEM of the FIB-etched RGO microball, as shown in Fig. S3 (c) and (d).

**Figure 3:**
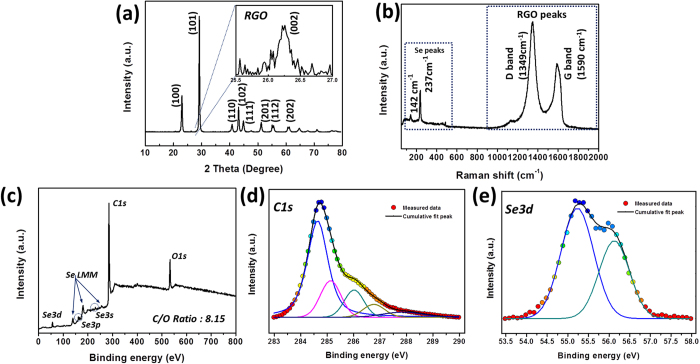
Structural characteristics of G–SeHMs (**a**) XRD pattern, (**b**) Raman spectrum, (**c**) full-scale XPS spectrum, (**d**) deconvoluted C1s XPS spectrum and (**e**) deconvoluted Se3d XPS spectrum.

Figure 3 shows the structural characteristics of G–SeHMs by X-ray diffraction (XRD), Raman spectroscopy and X-ray photoelectron spectroscopy (XPS). Figure 3(a) shows the XRD pattern of G–SeHMs. The intensities and positions of the indexed peaks, corresponding to the Se encapsulated within the graphene microballs, exactly coincided with the trigonal structure (Se_8_) [JCPDS 06-0362]^19^,^20^, indicating that the Se particles do not undergo a structural change or deterioration during spray-drying. Furthermore, a weak, broad signal was observed at approximately 26°, corresponding to the (002) plane of the RGO sheets^14^,^18^. As shown in in the Raman spectrum in Fig. 3(b), four dominant peaks were observed. The Se particles exhibited two characteristic peaks at 142 and 237 cm^−1^, corresponding to the trigonal crystal structure of Se_8_ molecules^6^,^21^. The D band, corresponding to structural defects and imperfections, and the G band, corresponding to the graphitic carbon on the RGO sheets, were observed at 1349 and 1590 cm^−1^, respectively; their relative intensities indicated that a high degree of carbonaceous material is graphitised^22^,^23^. Figure 3(c) shows the results obtained from the full-scale XPS analysis of G–SeHMs, providing information regarding the chemical states of C and Se and the degree of reduction of the GO sheets. The peaks related to Se and the RGO sheets indicated that the Se particles and RGO sheets coexist in the hybrid material. In addition, the degree of chemical reduction of the GO sheets using hydrazine hydrate can be determined by the deconvolution of the C1s XPS peak in Fig. 3(d)^18^,^23^. The XPS profile significantly decreased because of the defunctionalisation of GO sheets via strong chemical reduction using hydrazine hydrate during spray-drying. As determined from full-scale XPS analysis, the C/O ratio of the hybrid microballs was 8.15, which is greater than that for GO sheets. Notably, the degree of chemical reduction by hydrazine hydrate during spray-drying was sufficiently high for permitting high electrical conductivity of the hybrid material. In addition, two binding energy peaks were observed at 55.1 and 56.2 eV, respectively, in the deconvoluted Se 3d XPS profiles in Fig. 3(e), attributed to the Se−Se chemical bonds in a cyclo-octa-structured Se particles encapsulated in graphene microballs.

**Figure 4:**
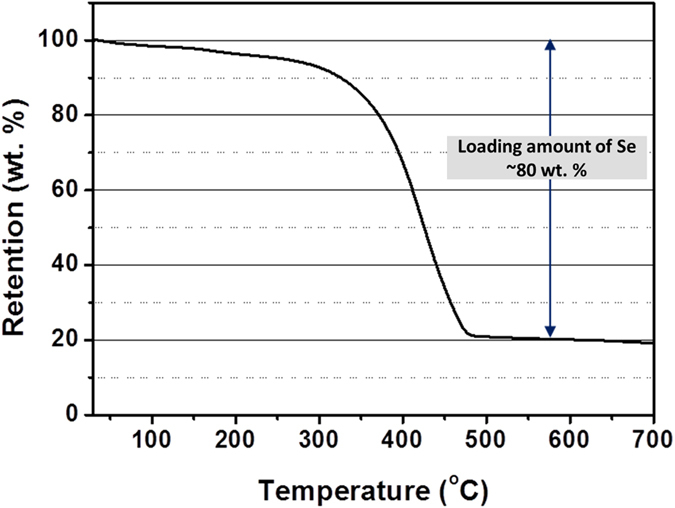
TGA profile of the G–SeHMs.

For determining the content of Se in G–SeHMs, thermogravimetric analysis (TGA) was performed under N_2_. As shown in Fig. 4, the content of Se in the hybrid microballs was as high as 80 wt%, which is significantly greater than that reported previously (Table 1). Further, the results obtained from comparative TGA performed with/without hydrazine hydrate (Fig. S4 in Supporting Information) confirmed that the chemical reduction of the GO sheets occurs during spray-drying.

**Figure 5:**
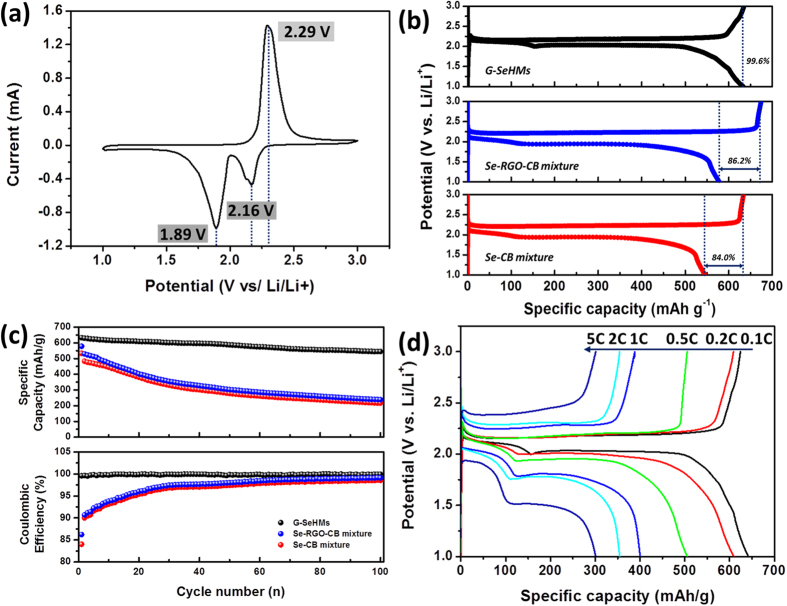
Electrochemical properties of the G–SeHMs as cathode materials for applications in lithium–selenium batteries. (**a**) Cyclic voltammograms at a scan rate of 0.1 mV s^−1^. Galvanostatic charge/discharge profiles of the G–SeHMs (top; black), Se–RGO–CB physical mixture (middle; blue) and Se–CB physical mixture (bottom; red) as control samples for comparison at a current density of 67.5 mA g^−1^, corresponding to a rate of 0.1 C. (**c**) Comparative study of cycling performance and Coulombic efficiencies of the G–SeHMs (black), Se–RGO–CB physical mixture (blue) and Se–CB physical mixture (red) after 100 cycles at 0.1 C. (**d**) Rate capabilities of the G–SeHMs at rates of 0.1–5 C.

The electrochemical properties of the hybrid material were evaluated by dissolving 1.0 M lithium bis-trifluoromethanesulfonimide (LITFSI) in tetraethylene glycol dimethyl ether (TEGDME) and 1,3-dioxolane (DOL) mixed in a volume ratio of 1:1 and using it as the electrolyte in a CR2032 coin cell. Figure 5(a) shows the cyclic voltammetry (CV) curves of the G–SeHMs at a scan rate of 0.1 mV s^−1^ after the first cycle. After the initial cathodic scan, a broad peak and a dominant peak were observed at 1.89 V and 2.16 V, respectively; these peaks correspond to the stepwise electrochemical reduction of Se to polyselenides and finally to Li_2_Se^8^,^9^,^24^. The subsequent anodic scan resulted in a strong oxidation peak at 2.29 V, corresponding to the electrochemical oxidation of Li_2_Se to polyselenides and elemental Se. This observation is in agreement with the CV profiles typically observed for the cathode materials used in Li–Se batteries. Furthermore, during discharging, two dominant plateaus were observed at 2.12 and 1.98 V, respectively, (Fig. 5(b)), corresponding to the reduction of Se to polyselenides. The G–SeHMs exhibited a specific capacity of 642 mA h g^−1^ at 0.1 C during the initial cycle (67.5 mA g^−1^ based on the weight of Se); this value corresponded to a Se electrochemical utilisation rate as high as 95.1%. Based on the weight of the hybrid materials, the specific capacity was 513.6 mA h g^−1^ for a Se content of 80 wt%. Because of the high loading amount of Se in the hybrid microball, the specific capacity based on the weight of the hybrid material was significantly greater than those of previously reported Se-based cathode materials (Table 1). Significantly, almost no overcharge was significantly observed during the initial cycle, indicating that the shuttling of Se is eliminated. On the other hand, the control samples of the Se–RGO–CB and Se–CB physical mixtures, which were prepared using an amount of carbon identical to that in the hybrid materials in Fig. 5(b), exhibited a lower specific capacity (578 and 532 mA h g^−1^ at 0.1 C for the Se–RGO–CB and Se–CB physical mixtures, respectively), indicating electrochemical utilisation rates of 85.6% and 78.8% and lower Coulombic efficiencies (CE = 86.2% and 84.0% for Se–RGO–CB and Se–CB, respectively); these values are dramatically less than that of G–SeHMs. These hybrid materials obtained from the large effective electrochemical surface area and electrical conductivity, values of which were greater than those of the Se–RGO–CB and Se–CB physical mixtures, might account for the higher electrochemical utilisation rates of the G–SeHMs. In addition, Fig. 5(c) shows the cycling performance and CE of G–SeHMS; for comparison, those of the Se–RGO–CB and Se–CB physical mixtures after 100 cycles at 0.1 C are shown. After 100 cycles, the specific capacity of the G–SeHMs was as high as 544 mA h g^−1^, indicating a capacity retention rate of 84.7% and a CE of 99.8% at 0.1 C. However, the Se–RGO–CB and Se–CB physical mixtures exhibited specific capacities of 211 and 188 mA h g^−1^, indicating capacity retention rates of 36.5% and 35.3%, respectively. As shown in Table S1 in Supporting Information, for the G–SeHMs as cathode-active materials, the CE was greater than 99% during 100 cycles, which was significantly greater than that for the two control samples, namely, Se–RGO–CB (1^st^ cycle: 86.2%, 100^th^ cycle: 99.0%) and Se–CB (1^st^ cycle: 84.0%, 100^th^ cycle: 98.5%). The G–SeHMs exhibited better cycling stability, attributed to the unique-structured graphene microball, which may suppress the diffusion of lithium polyselenide formed during discharging into the organic electrolyte. The G–SeHMs also exhibited good rate capability, in addition to good cycling performance, (Fig. 5(d)), exhibiting specific capacities of 610, 505, 400, 355 and 301 mA h g^−1^ at rates of 0.2 C, 0.5 C, 1 C, 2 C and 5 C, respectively. Even at a high rate of 5 C, the degree of electrochemical utilisation of Se in the G–SeHMs was 44.6%, which is very high for Li–Se batteries; such a high value is attributed to the unique morphological features of the G–SeHMs, which serve as confinement matrices for minimizing the dissolution of the polyselenides in the organic electrolyte and providing an electrically conductive path, thereby improving the electrochemical performance of the resulting Li–Se batteries. Notably, based on the weight of hybrid materials, the electrochemical performance is considerably better than that of previously reported Se-based cathode materials, attributed to high Se loading content (80 wt%) in hybrid materials (Table 1). Finally, as shown in Fig. S5 (Supporting Information), the volumetric capacity and specific area capacity were calculated for the G–SeHMs as cathode materials.

Inductively coupled plasma optical emission spectrometry (ICP-OES) was employed for measuring the amount of Se dissolved in the electrolyte at the end of the 100^th^ cycle at 0.1 C, which could provide quantitative information about the degree of dissolution of polyselenide. For ICP-OES measurements, 2032 coin cells were dissembled in an Ar-filled glove box, and their components (such as cathode, anode and separator) were then washed with 1,3-dioxolane. Then, the solution containing polyselenide species was collected and diluted with additional 1,3-dioxolane. After oxidizing the diluted solution using a concentrated aqueous HNO_3_ solution, the total selenium content was measured by ICP-OES. For comparison purposes, two coin cells composed of Se–RGO–CB and Se–CB mixtures, respectively, as control samples, were also disassembled and subjected to the same treatment after 100 cycles. From ICP-OES analysis, for the Se–RGO–CB–binder and Se–CB–binder, 60.9% and 64.1% of the total mass was lost to the ether-based organic electrolyte after 100 cycles, respectively. In contrast, for the electrode based on G–SeHM cathode materials, only 15.2% of the total Se mass was dissolved in the ether-based organic electrolyte after the 100^th^ cycle. After the cycling test, the SEM analysis of the electrode in G–SeHMs was conducted for investigating cycling-induced changes in the graphene micro-ball microstructure. As shown in Fig. S6 (Supporting Information), after the cycling test, the morphology of G–SeHMs was well retained. This result suggested that G–SeHMs exhibit good structural stability during electrochemical reactions. These results directly indicate that the dissolution of polyselenide species into the ether-based organic electrolyte is suppressed by the unique-structured RGO microball and that shuttling during charging/discharging is prevented, resulting in a better cycling stability and high CE for the Li–Se cell.

## Discussion

In this study, we fabricated morphologically unique graphene–selenium hybrid microballs in one step by aerosol microdroplet drying using commercial graphene oxide and selenium particles, which is a simple, scalable continuous process for manufacturing hybrid materials. Furthermore, we demonstrated that these hybrid materials can be used as cathode materials for applications of lithium–selenium secondary batteries, attributed to a high initial specific capacity (642 mA h g^−1^ at 0.1 C, corresponding to a Se electrochemical utilisation rate as high as 95.1%), good cycling stability (544 mA h g^−1^ after 100 cycles at 0.1 C; 84.5% retention) and high rate capability (specific capacity of 301 mA h g^−1 ^at 5 C). Notably, with high Se content in this hybrid cathode material, its electrochemical performance based on the weight of hybrid materials was comparable with that reported previously for Se-based cathode materials. Based on the abovementioned observations, these hybrid materials can be considered as a candidate electrode materials used in next-generation battery applications.

## Methods

### Preparation of a graphene oxide (GO)/Se suspension

First, a stable suspension of graphene oxide (GO), which was commercially obtained from Angstron Materials Inc., at 0.5 mg mL^−1^ was prepared in deionised (DI) water by probe-type ultrasonication for 1 h. In general, Se does not readily disperse homogeneously in DI water as its surface is hydrophobic. Thus, for obtaining a stable colloidal Se suspension in DI water, nonionic surfactant Triton X-100 (Sigma Aldrich) was used. Se particles at a 0.5 mg mL^−1^ concentration were dispersed in DI water by probe-type ultrasonication. Next, 3 mL of Triton X-100 was added to 1 L of this colloidal Se suspension. Finally, the mixture was continuously stirred using a magnetic stirrer for ensuring a homogeneous, stable suspension.

### Fabrication of G–Se hybrid microballs by spray-drying

First, Se and GO suspensions were mixed in a volume ratio of 3:1. Second, 10 mL of hydrazine hydrate (Sigma Aldrich), serving as the reducing agent, was slowly added dropwise to the mixture prepared in the first step. Next, the Se/GO/hydrazine hydrate suspension was maintained at 5 °C for inhibiting the reduction of GO to reduced GO (RGO) before spray-drying. Subsequently, a commercial spray dryer (B-290, Buchi) was utilised to form G–Se hybrid microballs from a stable Se/GO/hydrazine hydrate precursor suspension. During spray-drying, the suspension was injected at a feeding rate of 250 mL h^−1^ and atomised using a spray nozzle, generating aerosol microdroplets. These aerosol microdroplets were sprayed downwards towards a heated zone at 200 °C, which is significantly greater than the boiling point of water. As the aerosol microdroplets passed through the heated zone, the water in the microdroplets evaporated, and the chemical reduction of the nonconductive GO sheets occurred, resulting in conductive RGO sheets. Once the water completely evaporated, the Se particles were encapsulated in the RGO microballs. Subsequently, the final product was washed with ethanol and acetone for removing the residual Triton X-100.

### Characterisation

The microstructure of the microballs was examined by SEM (JSM-7001F, JEOL, Ltd.), TEM (CM200, Philips) and HR-TEM (JEM-2100, JEOL, Ltd.), while elemental mapping was carried out by EDX (X-MaxN, Oxford instruments). XRD (DMAX-2200, Rigaku) patterns were recorded at room temperature using Cu-Kα radiation (λ = 1.54056 Å) at a scan rate of 1° min^−1^, and the scans were performed at 0.04° intervals for 2θ values of 5°–80°. In addition, XPS measurements were conducted using an Omicron ESCA Probe (Omicron Nanotechnology) with monochromated Al-Ka radiation (hν = 1486.6 eV). Raman spectra (Jobin-Yvon LabRAM HR) were recorded at room temperature utilizing a conventional backscattering geometry and a liquid-nitrogen-cooled charge-coupled device multichannel detector. An argon-ion laser with a wavelength of 514.5 nm was utilised as the light source. The thermal properties of the G–Se hybrid microballs were determined using a thermogravimetric analyser (STA409 PC) under N_2_. Thermogravimetric analysis was carried out from room temperature to 1000 °C at a heating rate of 10 °C min^−1^. ICP-OES analysis was performed using a Thermo Scientific ICAP 6300 Duo View Spectrometer.

### Electrochemical characterisation

A CR2032 coin cell was fabricated by sandwiching a porous polypropylene separator (Celgard 2400) with a lithium metal foil anode in an Ar-filled glove box; subsequently, this coin cell was used for the characterisation of the room-temperature electrochemical properties of the hybrid material. The cathode consisted of a mixture of 80 wt% G–Se hybrid microballs as the working material, 10 wt% carbon black as the conducting agent and 10 wt% sodium alginate as the binder, which were dissolved in DI water to form a slurry. A 30 um thick doctor’s blade was used for uniformly coating the slurry onto an Al foil current collector, which was then dried at 100 °C for 24 h, followed by roll-pressing to a thickness of 20 μm. Each working electrode had an area of 1.13 cm^2^ (punched into discs with a diameter of 12 mm), and the amount of active material in the electrodes was approximately 2–3 mg cm^−2^. An organic electrolyte was prepared by dissolving 1 M LITFSI in a mixture of tetraethylene glycol dimethyl ether and 1,3-dioxolane mixed in a volume ratio of 1:1. Cyclic voltammetry and galvanostatic charge/discharge tests were performed using a potentiostat/galvanostat (VMP3, Princeton Applied Research) in the range 1.0–3.0 V.

## Additional Information

**How to cite this article**: Youn, H.-C. *et al*. Graphene–Selenium Hybrid Microballs as Cathode Materials for High-performance Lithium–Selenium Secondary Battery Applications. *Sci. Rep*. **6**, 30865; doi: 10.1038/srep30865 (2016).

## Supplementary Material

Supplementary Information

## Figures and Tables

**Table 1:** Comparison of the electrochemical performance in this study with that of previously reported cathode materials for Li–Se battery applications.

Sample	Se content in the composite^a^	Specific capacity based on the weight of selenium (C_Se_)	Specific capacity based on the weight of composite (C_comp._)^b^	Cycling stability^c^	Rate capability^c^	Ref.
Nanoporous Se	100 wt%	338 mAh/g at 0.1 A/g	338 mAh/g at 0.1 A/g	206 mAh/g	—	*Chem. Commun*. **49**, 11515 (2013)
Se/CMK-3	49 wt%	670 mAh/g at 0.1 A/g	328 mAh/g at 0.1 A/g	153 mAh/g	153 mAh/g at 5 C	*Angew. Chem. Int. Ed*. **52**, 8363 (2013)
Se/mesoporous carbon	30 wt%	480 mAh/g at 0.25 C	144 mAh/g at 0.25 C	144 mAh/g	66 mAh/g at 5 C	*ACS Nano* **7**, 8003 (2013)
Se/microporous carbon	51 wt%	895 mAh/g at 0.1 C	456 mAh/g at 0.1 C	127 mAh/g	—	*J. Mater. Chem. A* **2**, 17735 (2014)
Se/N-containing porous carbon	56 wt%	636 mAh/g at 0.5 C	356 mAh/g at 0.5 C	185 mAh/g	244 mAh/g at 2 C	*J. Mater. Chem. A* **2**, 12255 (2014)
Se/porous carbon aerogel	56 wt%	587 mAh/g at 0.5 C	329 mAh/g at 0.5 C	235 mAh/g	169 mAh/g at 2 C	*J. Power sources* **267**, 394 (2014)
Se/porous carbon bubble	50 wt%	691 mAh/g at 0.1 C	346 mAh/g at 0.1 C	303 mAh/g	216 mAh/g at 1 C	*Nanoscale*, **6**, 12952 (2014)
Se/C (PAN)	55 wt%	348 mAh/g at 0.05 A/g	191 mAh/g at 0.05 A/g	149 mAh/g	187 mAh/g at 0.5 A/g	*RSC Adv*. **4**, 9086 (2014)
Se/TiO_2_	71 wt%	481 mAh/g at 0.1 C	342 mAh/g at 0.1 C	112 mAh/g	—	*Solid State Ionics* **260**, 101 (2014)
Se/MCN-RGO paper	62 wt%	655 mAh/g at 0.1 C	406 mAh/g at 0.1 C	352 mAh/g	170 mAh/g at 3 C	*Adv. Funct. Mater*. **25**, 455 (2014)
Graphene–Se/CNT thin film	30 wt%	400 mAh/g at 0.1 C	120 mAh/g at 0.1 C	94 mAh/g	24 mAh/g at 1C	*J. Power Sources* **263**, 85 (2015)
Se/MWCNT	54 wt%	560 mAh/g at 0.1 A/g	302 mAh/g at 0.1 A/g	232 mAh/g	151 mAh/g at 1.2 A/g	*J. Mater. Chem. A* **3**, 555 (2015)
Graphene–selenium hybrid microball	80 wt%	642 mAh/g at 0.1 C	514 mAh/g at 0.1 C	435 mAh/g	241 mAh/g at 5 C	*This study*

^a^Se content was determined by thermogravimetric analysis (TGA) in previous studies, as well as in this study.

^b^Specific capacity (C_comp_) based on the weight of composite was calculated by following equation. (C_comp_ = specific capacity (C_Se_) based on the weight of Se × Se content in the composite).

^c^The values obtained for cycling stability and rate capability were calculated by the weight of the composite.
