# Supplementary material for: Graphene–Selenium Hybrid Microballs as Cathode Materials for High-performance Lithium–Selenium Secondary Battery Applications
Source: Sci Rep. 2016 Aug 2;6:30865. doi: 10.1038/srep30865 (PMC4969586; doi:10.1038/srep30865)
Supplement: Supplementary Information [file srep30865-s1.doc]

Graphene–Selenium Hybrid Microballs as Cathode Materials for High-performance Lithium-Selenium Secondary Battery Applications

Hee-Chang Youn ^a^, Jnu Hui Jeong ^a^, Kwang Chul Roh ^b^,*, and Kwang-Bum Kim ^a^,*

^a^Department of Materials Science and Engineering, Yonsei University, Seoul 120-749, Republic of Korea

^b^Energy and Environmental Division, Korea Institute of Ceramic Engineering and Technology, Jinju 660-031, Republic of Korea

**
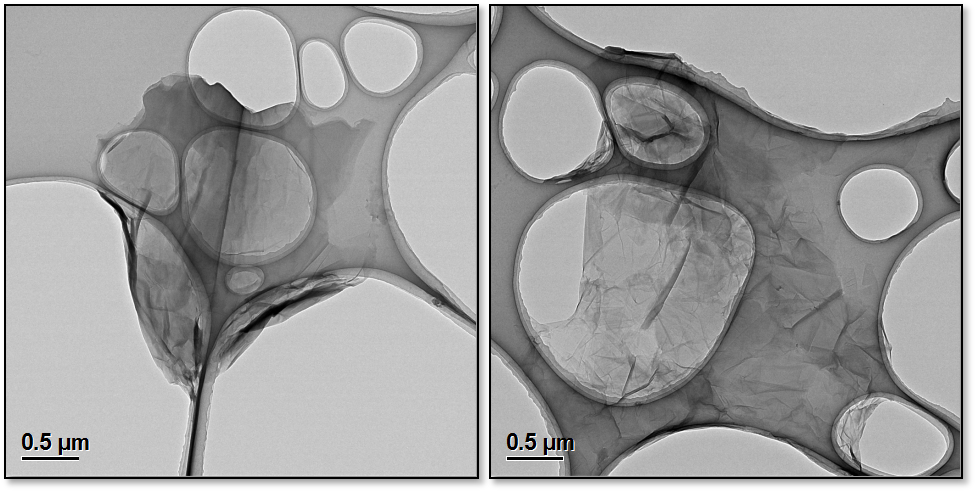
**

**Figure S1.** High-resolution TEM images of the GO sheets, with a size of a few micrometres, used in this study.


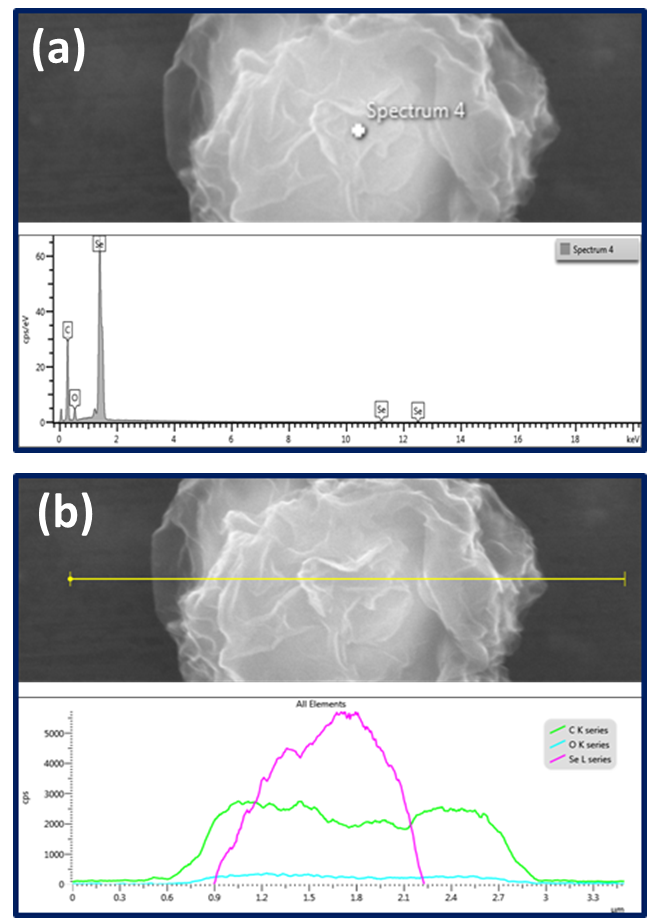


**Figure S2.** (a) Point- and (b) line-scanning elemental spectra of the G–Se hybrid microballs.


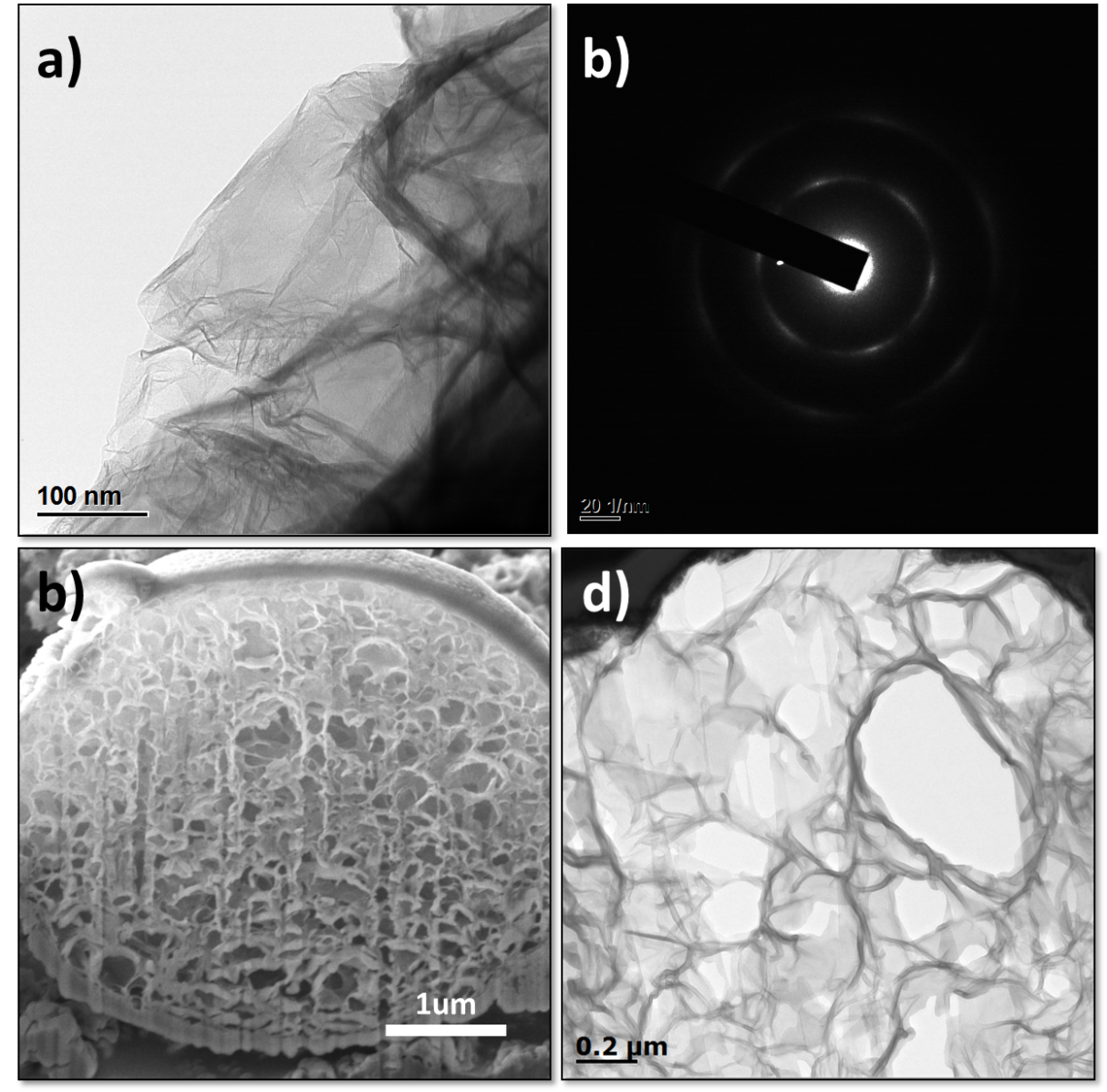


**Figure S3.** (a) High-magnification HR-TEM image and (b) SAED pattern of G–SeHMs. (c) Cross-sectional (c) SEM and (d) TEM of FIB-etched RGO microball without Se particles

As shown in the high-magnification TEM image in **Figure S3 (a)**, ultrathin layers were observed at the edges, suggesting the presence of a single or a few layers of graphene in the RGO microball. Furthermore, as shown in the SAED pattern in **Figure S3 (b)**, well-defined diffraction spots were observed in a hexagonal pattern, indicating that the basal plane in the RGO microball mostly consists of sheets of a single or a few layers comprising honeycomb carbon networks. For investigating the structural features of the interior of the RGO microball, a synthetic process identical to that utilised for G–SeHMs was employed for preparing an RGO microball without selenium particles, and the microball was characterised by cross-sectional SEM and TEM of the FIB-etched RGO microball in **Figure S3 (c)** and **(d)**. The inter-connected RGO sheets were evenly dispersed not only near the surface of the microball but also within the microball. The interconnected, complex internal structure of the RGO sheets can provide an effective confinement matrix for suppressing the dissolution of polyselenide into the organic electrolyte during the redox reaction and can provide electron pathways, both at the surface and within the interior of the microball, for a better electrochemical reaction.

**
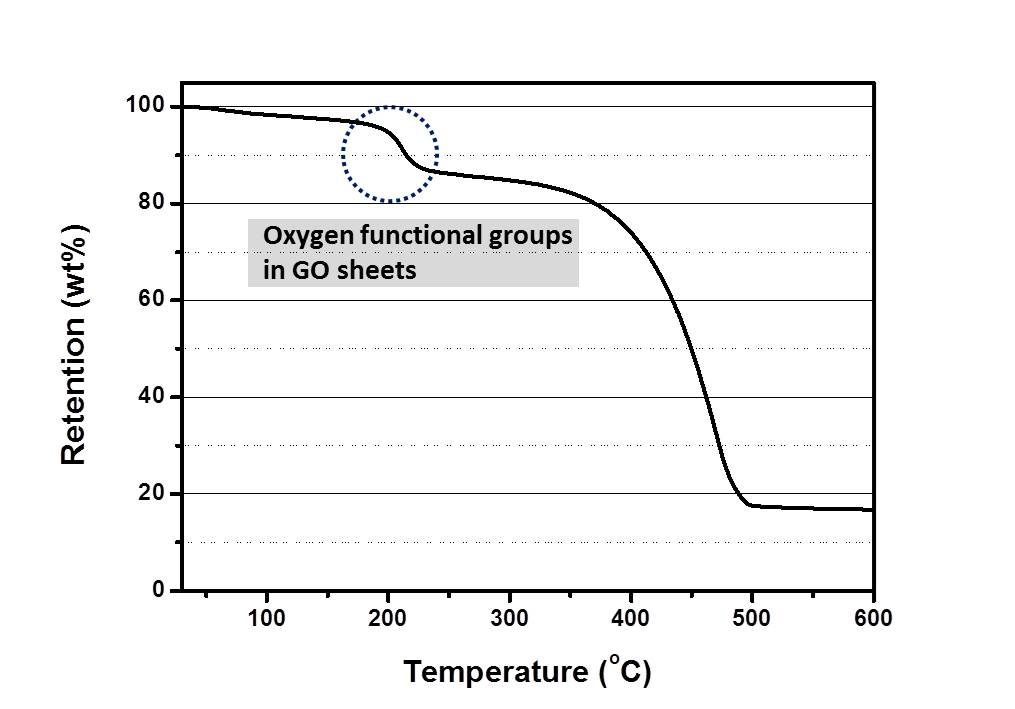
**

**Figure S4.** Thermogravimetric analysis curve of GO–Se hybrid microballs prepared by the same method as that employed for the G–Se hybrid microballs; the difference was that hydrazine hydrate was not employed as a chemical reducing agent, resulting in the retention of oxygen functional groups on GO sheets and no occurrence of the chemical reduction of GO.


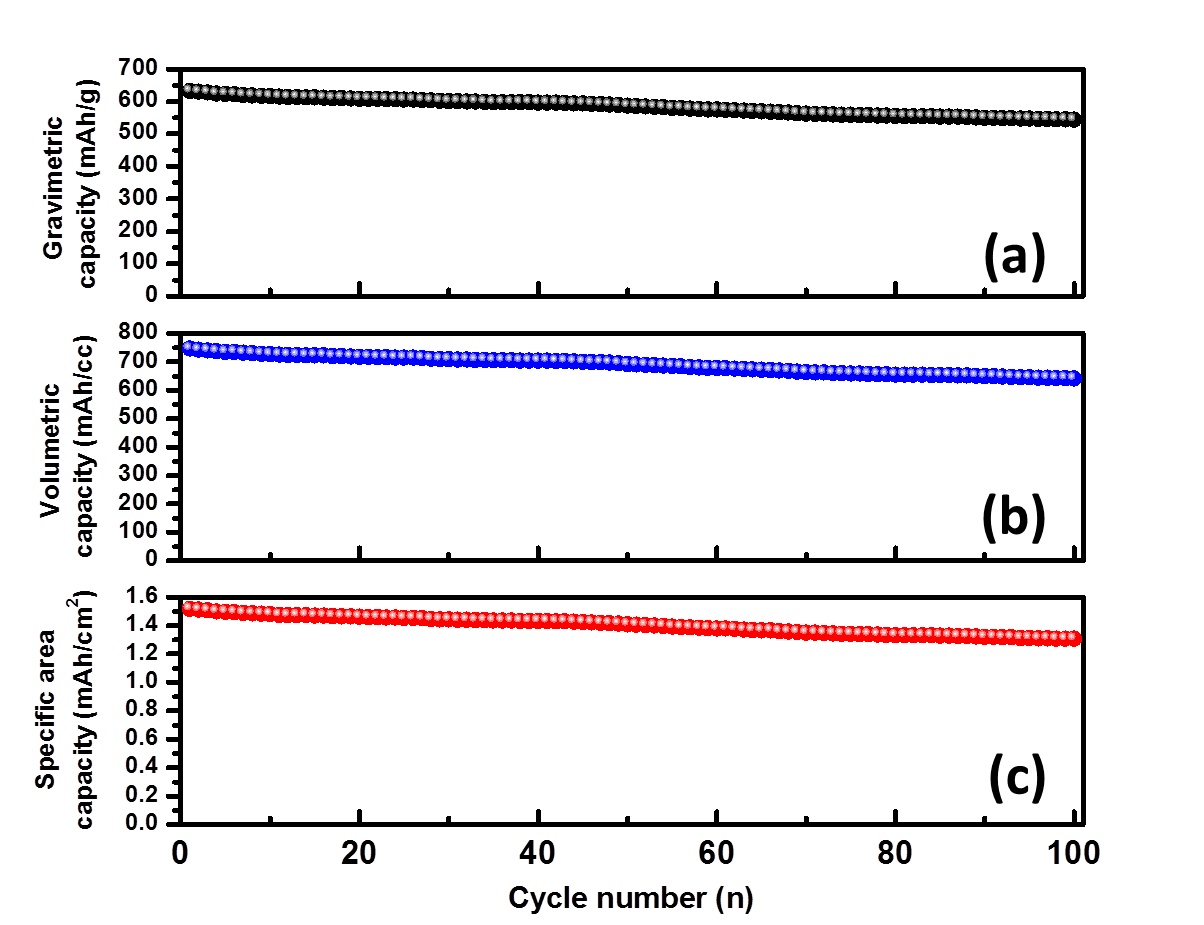


**Figure S5.** Cycling stability based on (a) gravimetric capacity, (b) volumetric capacity and (c) specific area capacity of G–SeHMs as cathode materials.

For calculating the volumetric capacity and specific area capacity of the G–SeHMs, the electrode density and selenium mass loading per unit area of the G–SeHM electrode were first obtained from the average value (10 electrode samples) of the weight of active materials (2.67 mg), thickness of the active material (20 μm) and electrode area (Φ12; 1.13 cm^2^).

The electrode density of the G–SeHM electrode was calculated using *eq. R1.1* as follows:


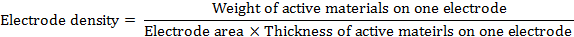

 (eq. 1.1)


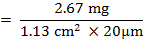

= 1.18 g/cc

The selenium mass loading per unit area of the G–SeHM electrode is calculated using *eq. 1.2* as follows:


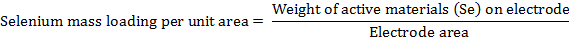

 (eq. 1.2)


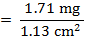

= 0.0015 g/cm^2^

The volumetric capacity and specific area capacity are calculated using eqs. 1.3 and 1.4, respectively, as follows:

**Volumetric capacity (mAh/cc)**

= Gravimetric capacity (mAh/g) × Electrode density (g/cc) (eq. 1.3)

**Specific area capacity (mAh/cm^2^)**

= Gravimetric capacity (mAh/g) × Selenium mass loading per unit area (g/cm^2^) (eq. 1.4)

The gravimetric capacities shown in **Figure R4 (a)** were converted to volumetric capacities in **Figure R4 (b)** and specific area capacities in **Figure** **R4 (c)**, based on their electrode densities (1.18 g/cm^3^) and selenium mass loading amount per electrode unit area (0.0015 g/cm^2^). The initial volumetric capacity and specific area capacity of the G–SeHMs are calculated to be as high as 746 mAh/cc and 0.95 mAh/cm^2^, respectively. Currently, experiments for the enhancement of electrode density and active material mass loading for achieving a better volumetric capacity and specific area capacity without sacrificing gravimetric capacity, rate capability and cycling stability are underway in our laboratory.


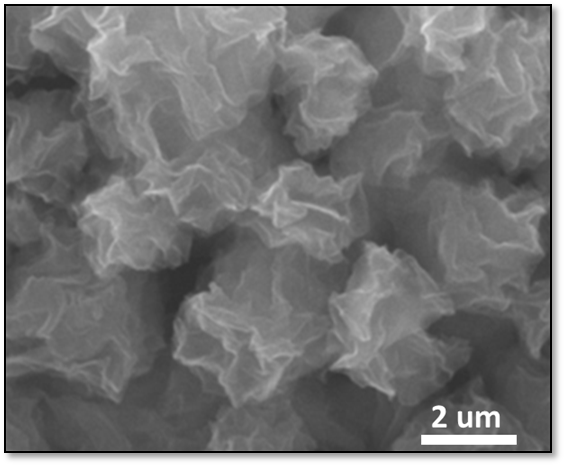


**Figure S6** SEM image of an electrode of the G–SeHMs after 100 cycles

**Table S1** Comparative study on the Coulombic efficiency of three cathode electrodes.

| Cycle number | G–SeHMs | | | Se–RGO–CB mixture | | | Se–CB mixture | | |
| --- | --- | --- | --- | --- | --- | --- | --- | --- | --- |
|  | CC | DC | CE | CC | DC | CE | CC | DC | CE |
| 1^st^ | 644 | 642 | 99.6 | 671 | 578 | 86.2 | 633 | 532 | 84.0 |
| 5^th^ | 633 | 632 | 99.8 | 556 | 513 | 92.2 | 512 | 469 | 91.6 |
| 10^th^ | 627 | 625 | 99.7 | 503 | 472 | 93.8 | 477 | 445 | 93.2 |
| 20^th^ | 618 | 617 | 99.8 | 420 | 403 | 95.9 | 392 | 374 | 95.4 |
| 50^th^ | 597 | 596 | 99.8 | 308 | 302 | 98.1 | 285 | 278 | 97.4 |
| 100^th^ | 545 | 544 | 99.8 | 213 | 211 | 99.0 | 191 | 188 | 98.5 |

* CC: Charge capacity (mAh/g); DC: Discharge capacity (mAh/g); CE: Coulombic efficiency (%)

As shown in **Table S1**, the Coulombic efficiency of the G–SeHMs as the cathode-active material was greater than 99% during 100 cycles, which was significantly higher than that for the two control samples, namely, Se–RGO–CB (1^st^ cycle: 86.2%, 100^th^ cycle: 99.0%) and Se–CB (1^st^ cycle: 84.0%, 100^th^ cycle: 98.5%). We believe that the good cycling stability and Coulombic efficiency of G–SeHMs as cathode material is attributed to the unique-structured RGO microball, which effectively suppresses the diffusion of lithium polyselenide formed during discharging into the organic electrolyte.
